# Knowledge Discovery Using Topological Analysis for Building Sensor Data †

**DOI:** 10.3390/s20174914

**Published:** 2020-08-31

**Authors:** Manik Gupta, Nigel Phillips

**Affiliations:** 1 Department of Computer Science and Information Systems, BITS-Pilani, Hyderabad 500078, India; 2 Department of Computer Science and Informatics, London Southbank University, London SE1 0AA, UK; nigelpphillips@gmail.com

**Keywords:** BEMS, HVAC, Internet of Things, computational topology, Q-Analysis, knowledge discovery, data mining

## Abstract

Distributed sensor networks are at the heart of smart buildings, providing greater detail and valuable insights into their energy consumption patterns. The problem is particularly complex for older buildings retrofitted with Building Energy Management Systems (BEMS) where extracting useful knowledge from large sensor data streams without full understanding of the underlying system variables is challenging. This paper presents an application of Q-Analysis, a computationally simple topological approach for summarizing large sensor data sets and revealing useful relationships between different variables. Q-Analysis can be used to extract novel structural features called Q-vectors. The Q-vector magnitude visualizations are shown to be very effective in providing insights on macro behaviors, i.e., building floor behaviors in the present case, which are not evident from the use of unsupervised learning algorithms applied on individual terminal units. It has been shown that the building floors exhibited distinct behaviors that are dependent on the set-point distribution, but independent of the time and season of the year.

## 1. Introduction

The rise of Internet of Things (IoT) has promoted a new class of applications that can help improve process control as well as enable intelligent decision making. The ability to add sensors capable of monitoring activity in complex systems with real-time reporting via the Internet presents many opportunities for effective automated control without the need for human involvement. In many situations, such monitoring adds a timeliness and finer data granularity allowing improvements in well-understood situations [1]. There is a much larger class of possible applications where the dynamics are not yet understood and the potential for gains is far greater [2]. Retrofitted Building Energy Management Systems (BEMS) are a good example of this class of large scale, distributed sensor based IoT applications. This paper presents the first application of a topological approach called Q-Analysis to such sensor data, that the author is aware of, and shows that Q-Analysis complements the existing exploratory data analysis methods and promotes technological knowledge discovery by exploring the connections and dependencies between data.

**Figure 1:**
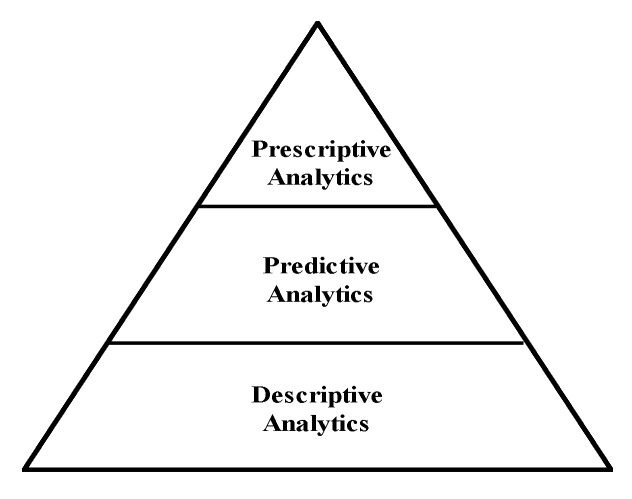
Hierarchy of descriptive, predictive and prescriptive analytics.

BEMS are a key driver of IoT deployments in both existing and future buildings. BEMS are comprehensive, IT-based platforms that extend the capabilities of sensing, control, and automation hardware to direct automated and/or manual improvements and system operations utilizing a large volume of multivariate time series data retrieved from various Heating, Ventilation and Air-Conditioning (HVAC) components [3]. BEMS record a wide variety of energy related data at regular intervals from locations throughout a building generating large volumes of time series data with the intention of improving occupant comfort and reducing energy costs. This situation can be described as data rich, knowledge poor. To leverage the rich flows of data that BEMS provide, data analytics techniques must be developed that model the data so that useful information can be extracted [4,5,6]. Analytical techniques can be roughly divided into three classes: descriptive, predictive and prescriptive, that form a hierarchy as shown in Figure 1 [7]. Descriptive techniques are needed to structure the data, so that predictive techniques can be effective, and predictive techniques need to be effective before corrective actions can be prescribed.

The focus of the current paper is specifically on a HVAC component known as a terminal unit (TU). A TU is a specific subcomponent of HVAC systems that is responsible for the final delivery of comfort inside built environments. Usually, there are hundreds to thousands of TUs deployed within a single building and poorly controlled or faulty TUs can be responsible for significant energy wastage and occupant discomfort in buildings. For example, a faulty TU can signal a false heating demand to the boiler, causing the boiler and ancillary equipment to activate and begin distributing hot water, unnecessarily overheating other room spaces. In real life scenarios, TUs can suffer from a number of potential issues owing to poor control, poor sensor location, variable or unachievable set points, out of hours operation, competition from nearby Tus, etc. This highlights the need to identify anomalous TU behaviors so as to save energy as well as avoid occupant discomfort without requiring any human intervention [8]. The primary goal of this work is to demonstrate an effective method to remotely analyze, categorize different TU behaviors and further identify individual floors as exhibiting either a dynamic or stable behavior based on the location of anomalous TUs from BEMS data streams. The state of each TU is known at regular time intervals including whether it is consuming heating or cooling power and the control temperature in relation to the comfort zone: the set point (target temperature) and the dead band upper and lower bounds within which the control temperature is permitted to vary without any corrective action. However, none of the above characteristics provide any definitive evidence of occupant comfort being met, although it is assumed that if the set point is adjusted, it implies that comfort is not being met.

Unsupervised clustering techniques such as k-means [9] and principal component analysis [10] have been used in previous works [11]. The author of [12] carried out a study to categorize TUs behaviors, but this proved to be ineffective with BEMS data because of the lack of explanation for the potential reasons for TU malfunctioning and the lack of clustering validation for different TU cluster behaviors without appropriate expert building engineer knowledge. Additionally, the categorization of individual floor behaviors based on TU behaviors was also not possible. This motivated the application of computational topology [13,14,15] as an alternative approach to categorize TU data based on the shape within a space of possibilities. 

Topological methods have recently risen into prominence to extract information from high dimensional datasets for various data science applications and hold much promise to provide useful insights into system behavior [16,17]. Topological techniques capture the shape characteristics of the data via invariants such as the Euler characteristic, and equivalence classes such as homotopy and homology [18,19,20]. Geometrical structures known as simplicial complex can be generated from discrete point cloud data and used to reconstruct and study the topology of the underlying object. It enables examination of higher order relationships as opposed to pairwise relationships examined in clustering techniques, and interesting topological features can be extracted for data summarization purposes [15]. They are particularly helpful for analysis of datasets that exhibit rich underlying structures and involve complex interactions. There have been a few recent applications of topological analysis to aviation data, gene expression data and digital epidemiology [21,22] however there is still a huge gap in adoption of topological techniques from the data mining community. Hence, this work attempts to fill in the gap with application of topological methods to building data and highlight their effectiveness as an alternative to traditional data analysis methods.

One of the key contributions of the paper is to present a first application of Q-Analysis, an applied topological approach that focuses more on connectivity than shape for generating a summary of BEMS data from large sets of distributed sensor data. The technique is applied to TU data and is found to be more effective at extracting useful summary information and identifying relationships between involved variables. Both novel feature extraction and data visualizations based on application of Q-Analysis have been proposed and have shown to provide greater insight into individual building floor behaviors.

A brief outline of the paper is as follows. Section 2 summarizes the mathematical foundations of topology and Q-Analysis. Section 3 presents the methodology adopted and describes Q-Analysis based analytical pipeline for BEMS data. Results are presented and discussed in depth in Section 4. Section 5 draws conclusions on the paper and discusses the future research directions.

## 2. Topology and Q-Analysis

In simple terms, topology is the study of the shape of objects subject to operations such as stretching and twisting and it has deep roots within the field of algebraic topology. This section introduces Q-Analysis and its relationship to the wider key aspects of topology.

### 2.1. Topology

Topology gives a precise formal meaning to the notion of proximity and continuity defined by nested systems of open neighborhoods, it defines nearness without the need of a distance metric [16]. A topology is a collection of open sets in a space *X*. 

Definition (Topology): Formally a topology *T* on a set *X* is a collection *T* of subsets of *X* such that

1. *∅* and *X* are in *T*.
2. The union of any sub collection of *T* is in *T*.
3. The intersection of any finite sub collection of *T* is in *T*.

A set *X* for which a topology *T* has been specified is called a topological space [17].

Definition (Open sets): If *U* is a subset of *X*, with topology *T* and *U* belonging to the collection *T*, then *U* is an open set if

1. *X* and *∅* are open.
2. Arbitrary unions of open sets are open.
3. Finite intersections of open sets are open.

As a subject, topology is concerned with various equivalence relations between spaces that persist even when the spaces are deformed. Two spaces are homotopy equivalent if one can be continuously deformed into another without breaking or tearing; and two spaces are homologically equivalent if they contain the same number of components, holes, voids, etc. Two equivalent spaces are not necessarily exactly the same, but two nonequivalent spaces are definitely different. Hence, topology is good for *characterization*: classifying spaces by identifying global characteristics; *continuation:* distinguishing between true features, and those that are the product of noisy data; *integration*: converting local data into global properties [16].

Dowker [23] has shown how any relation between well-defined sets has an associated homology through the abstract simplicial complexes the relation induces on each set. Atkin [24,25] exploited this to develop an applied topological approach known as Q-Analysis for tackling high dimensional problem spaces in the social sciences and urban planning applications [26,27]. Q-Analysis captures structure through the ‘threads of connection’ between the elements of the sets. It is more informal and less computationally demanding than a full homology approach with high dimensional data and has proved useful at distinguishing system states in the BEMS data.

### 2.2. Simplicial Complex

#### 2.2.1. Simplices

Definition (Simplex): Given a geometrically independent set of points {*a*_0_, …, *a_n_*} in R^n^. The n-simplex spanned by *a*_0_, …, *a_n_* is the set of all points in R^n^ such that

(1) $$x=\overset{n}{\underset{i=0}{\sum }}t_{i}a_{i},\;\;where\;\overset{n}{\underset{i=0}{\sum }}t_{i}=1\;and\;t_{i}\geq 0\;for\;all\;i.$$

**Figure 2:**
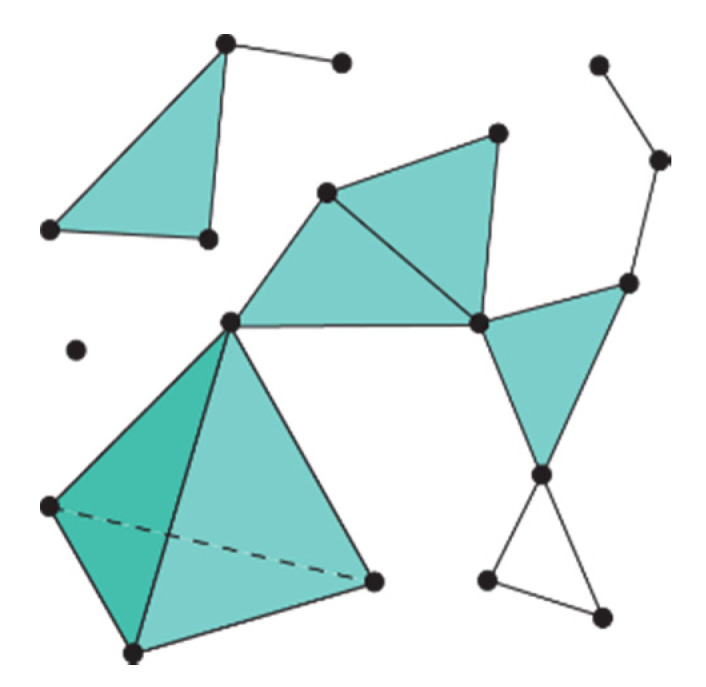
Simplicial Complex.

Points are geometrically independent if they are in a general position, i.e., three points in R^2^ are independent if they do not fall on a straight line, four points in R^3^ do not fall on a plane, etc. A 0-simplex is a point, a 1-simplex is a straight line, a 2-simplex is a triangle, a 3-simplex is a tetrahedron and n-simplex (n > 3) is the higher dimensional equivalent (see Figure 2 [28]).

#### 2.2.2. Abstract Simplicial Complex

The notion of a simplex can be generalized to any collection of sets as follows:

Definition (Abstract Simplicial Complex): An Abstract Simplicial Complex is a collection *S* of finite nonempty sets, such that, if *A* is an element of *S*, then so is every nonempty subset of *A*. 

If a set *X* can be mapped to an abstract simplicial complex, then the complex and *X* define a topological space. Further, any relation between two well-defined sets induces a topology on each set provided by the cover one set makes of the other defining a pair of simplicial complexes [23]. If *R* is a relation between sets *A* and *B*, *A R B*, then if *a* ∈ *A R* {*b*_1_, *b*_2_, …, *b_n_*}, where *b*_1_, *b*_2_, …*b_n_* ∈ *B,* then *A* defines a (n − 1)-simplex. 

Atkin [24] developed Q-Analysis with this connectivity in mind based on the work of Dowker [23] on the homology groups of a relation to derive a number of useful metrics based on simple integer calculations. It introduces the notion of q-equivalence, which provides an easily computable equivalence based on simplex faces that provide a metric free hierarchical clustering of set members and Q-vectors, which provide a metric for comparing global structure and eccentricity for characterizing local structure. Both q-equivalence and Q-vectors are described in further detail in the following section.

### 2.3. Q-Analysis

Atkin developed a theory of pseudo-homotopy called shomotopy [25,29]. The original presentation was shown to be flawed, but has been put on a firm mathematical footing using Galois Theory [30,31,32].

A relation between two sets *A* and *B* is represented by an incidence matrix *R* with |*A*| rows and |*B*| columns (where |*X*| indicates the cardinality of the set *X*), each cell *r_ij_* contains a 1 if the relation holds between *a_i_* ∈ *A* and *b_j_* ∈ *B* or 0 otherwise.

*R* can be used to define a relation from *A* to *B* and inverse relation *R*^−1^ from *B* to *A*. Say, for instance, if *A* is a set of documents and *B* is a set of words and the relation between *A* and *B* is ‘document contains word’, then the inverse relation is ‘word appears in document’. Similarly, if *A* is a set of sensors and *B* is a set of sensor states and the relation between *A* and *B* is ‘sensor exhibits state’ then the inverse will be ‘state is exhibited by sensor’. In the work presented in this paper, the set of TUs constitute one set and the states exhibited by the TUs constitute the other set and the incidence matrix created using these two sets is shown in Section 3.3.1. This will be explained further in Section 3.2 and Section 3.3. This relation from one set to another imposes structure on each set, which can be analyzed as an abstract simplicial complex as mentioned in previous Section 2.2. If two elements of *A* relate to the same n elements of *B,* then they share a q = (n − 1) dimensional face or are said to be q-near. 

#### 2.3.1. Calculating Equivalence Classes for Q-Analysis

Q-Analysis takes the incidence matrix *R* of a relation and creates a shared face matrix for each set by left and right multiplying by *R^t^* (the transposed of *R*). The shared face matrix indicates the ‘direct’ connections between pair of simplices. However, it also enables computing the q-equivalence i.e., the number of common vertices and dimension of the shared face by subtracting one from the number of common vertices. 

Definition (q-equivalence): If *a*_1_, *a*_2_ ∈ *A* are both related to the set {*b*_1_, *b*_2_, …, *b_n_*}, where *b*_1_, *b*_2_, …, *b_n_* ∈ *B*, then *a*_1_, *a*_2_ are q = (n − 1) connected. Components that are q-connected are members of the same q-equivalence class. 

Left multiplication of *R* by its transpose *R^T^R* is carried out to create a |*A*| × |*A*| matrix which counts the number of points each element a ∈ *A* has in common with every other element of *A*. Subtracting 1 from each entry gives the dimension of each shared face. Similarly, right multiplying *RR^T^* and subtracting 1 gives a |*B*| × |*B*| matrix of faces shared by elements of *B*. This leads to the creation of shared face matrices as shown in Section 3.3.2.

The diagonal of the matrix defines the largest face of each element. The maximum value of the diagonal defines the dimension of the simplicial complex *S*, dim(*S*) = K. This also represents the maximum q-level. An object is present at a q-level if it has a face greater than or equal to q. Two objects share an equivalence class if they share a q-face. 

#### 2.3.2. Calculation of Structure Vectors

Equivalence classes can be thought of as a way to cluster objects and the successive equivalence classes at lower levels form a hierarchy. In this way Q-Analysis provides a hierarchical clustering of objects without the need for a metric space. The equivalence classes impose a structure on the sets and Q-vector can be used as an objective measure to capture the structure exhibited by the data. Q-vector is the count of equivalence classes at each q-level and is formally defined as follows.

Definition (Q-vector): A Q-vector for a simplicial complex *S* is a vector (Q_k_, Q_k−1_, … Q_0_) where each Qi = the number of q-equivalence classes, where q = dim(Q_k_) [22].

Q-analysis can be used to reveal the q-connectivity lists at different q-levels, where the objects are q-connected if there is a chain of q-faces (q-chains) connecting them. The q-connectivity lists can be used to count the number of connected components at each level and corresponding Q-vectors can be generated as shown for BEMS data in Section 3.3.3. In this work, it is proposed to use Q-vector magnitudes as novel features representing the structure exhibited by the TUs and their corresponding states. The Q-vector provides a way to describe the global structure of the entire simplicial complex rather than individual equivalence classes. However, within an equivalence class the relationships can vary markedly and can give an idea about the local structure. For classes with cardinality greater than 2, membership of a class does not mean that each member shares a q-face with every other member, only that it shares a q-face with at least one other member. This means that two relations with identical Q-Vectors could have considerable variation at fine granularity. 

## 3. Methodology 

The methodology for application of Q-Analysis to BEMS data is divided into the following steps:

Step 1. Collection and evaluation of raw BEMS data.
Step 2. Filtering specific dataset groups followed by data preprocessing.
Step 3. Feature extraction over the preprocessed datasets to discover useful information.
Step 4. Visualizations to provide insights into the data.

**Figure 3:**
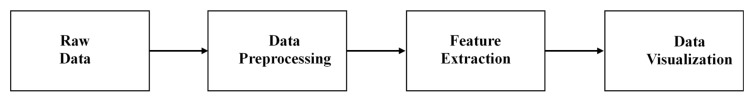
Data analytics pipeline.

Each of the steps as shown in Figure 3 are explained in more detail in the following subsections.

### 3.1. Description of BEMS Raw Data

The present case study is based on a building located in the city of London. The building has 17 floors and 731 TUs spread across the different floors. There are various data streams that are collected for a single TU and each of the data variables is described as follows:

1. Control temperature [°C] is the reported space temperature, frequently measured by each TU or in some cases a zone space temperature is used.
2. Set point temperature [°C] is the desirable control temperature for a TU by the operator or an administrator based on the current demand. Deadband [°C] is the temperature range between heating and cooling set points, within which heating or cooling equipment is not operated.
3. Average power is calculated from percentage valve demand i.e., the effort exerted by a heating or cooling valve. A nominal rated power of 1 kW has been assumed for all TUs and provides an estimate of the energy consumption of each TU.
4. Enabled signal indicates the hours of operation.
5. Temperature error [°C] is the deviation of control temperature from the desired set point settings.

**Figure 4:**
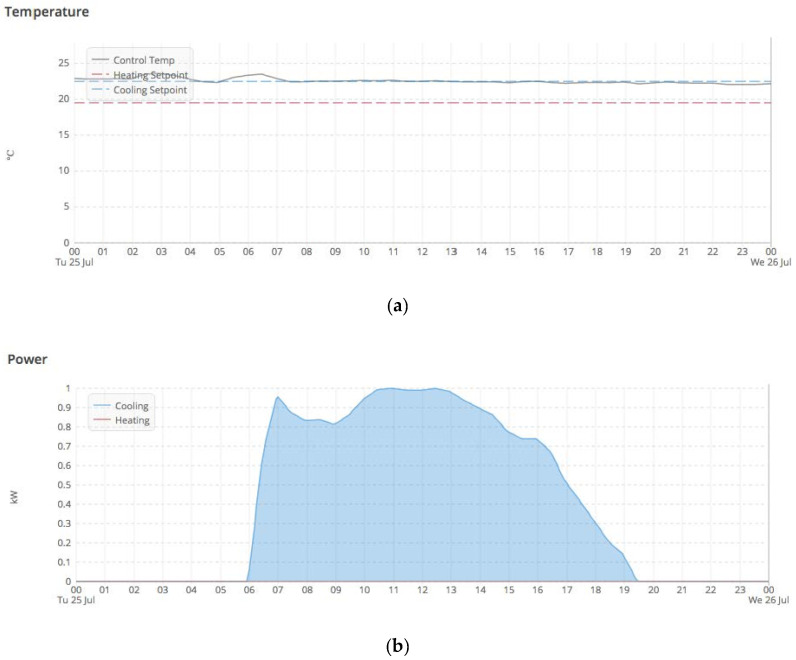
(**a**) Control temperature, heating and cooling set points (**b**) Cooling power consumption for a Terminal Unit.

There are many more HVAC variables collected via the BEMS, but the above-mentioned parameters are the most significant ones related to TUs, according to the domain experts—building engineers and managers—in the present case. Figure 4 represents the raw data that is obtained for a single TU located on one of the building floors on a particular day. Figure 4a shows the control temperature being maintained by the TU within the desired cooling set point (blue dotted line) and heating set point (red dotted line) settings. Figure 4b shows the cooling effort exerted (blue shaded area) or cooling power consumed by the TU. The TU is enabled within the operational hours from 6 am to 6 pm to maintain the control temperature within the set point limits.

### 3.2. Data Preprocessing

First, the TUs for a particular building are grouped by floor levels and then the data preprocessing is carried out for each TU. In order to represent the relations between the TUs and their state (corresponding to each data variable) at time T, the continuous data variables need to be converted to discrete intervals. The data preprocessing stage incorporates the following sub steps.

#### 3.2.1. Quantization of Continuous Variables to Ordinal Data

For a group of TUs, the subset of available data streams is selected and continuous measures are converted into discrete, ordinal ranges. For example: standard quartiles, such as the lower quartile, median and upper quartile ranges or user defined percentiles, such as very high (x > 80 percentile), high (60 < x ≤ 80 percentile), medium (40 < x ≤ 60 percentile), low (20 < x ≤ 40 percentile), very low (0 < x ≤ 20 percentile) can be chosen to divide the distribution of values for a particular variable. There are other possibilities, as well for dividing into ordinal ranges, depending on the application requirements.

For instance, the control temperature has been quantized into high, medium and low ranges. So, a TU for any given time interval, could either be in a low control temperature, medium control temperature or a high control temperature state. Similarly, the set point and temperature error has been quantized into high, medium and low ranges. For cooling and heating power, the data variables are divided into low, medium and high ranges. Additionally, there is a possibility that some of the TUs use maximum heating or cooling power. To accommodate this additional state exhibited by the TUs, maximum heating and maximum cooling power has been considered as a separate discrete range.

#### 3.2.2. Choice of Temporal Focus

The quantization can be carried out across different time frames—either across time intervals such as minutes, hours, days, weeks, months or regular intervals e.g., from 6 am to 9 am every Monday for 10 weeks. In the current study, a 12 hourly behavior period was studied for the different TUs and it was found that it was difficult to differentiate between different TU behaviors during that time interval, hence a shorter time interval was experimentally chosen for analysis—15 min—which provided a reasonable differentiation between TU behaviors. 

### 3.3. Feature Extraction

Once the initial data preprocessing is done, Q-Analysis can be carried out to create incidence matrices, q-equivalence classes and q-connectivity lists as specified in Section 2.3. Next the Q-vectors can be computed as unique features and can be further visualized to draw key data insights. The following subsections explain the creation of incidence matrix followed by the feature extraction using Q-Analysis in more detail.

#### 3.3.1. Creation of Incidence Matrix

The incidence matrix provides an analytical representation of the different state of each TU at time T. Each TU can be represented by a geometrical figure or convex polyhedron called a simplex. The vertices of a simplex are the states exhibited by that particular TU. In general, if a TU exhibits q + 1 states, its dimension is q. It is evident that there will be TUs that have one or more states in common with one another. These simplices form a connected, multidimensional space or structure called the abstract simplicial complex and it is the connectivity structure that Q-Analysis explores. 

**Figure 5:**
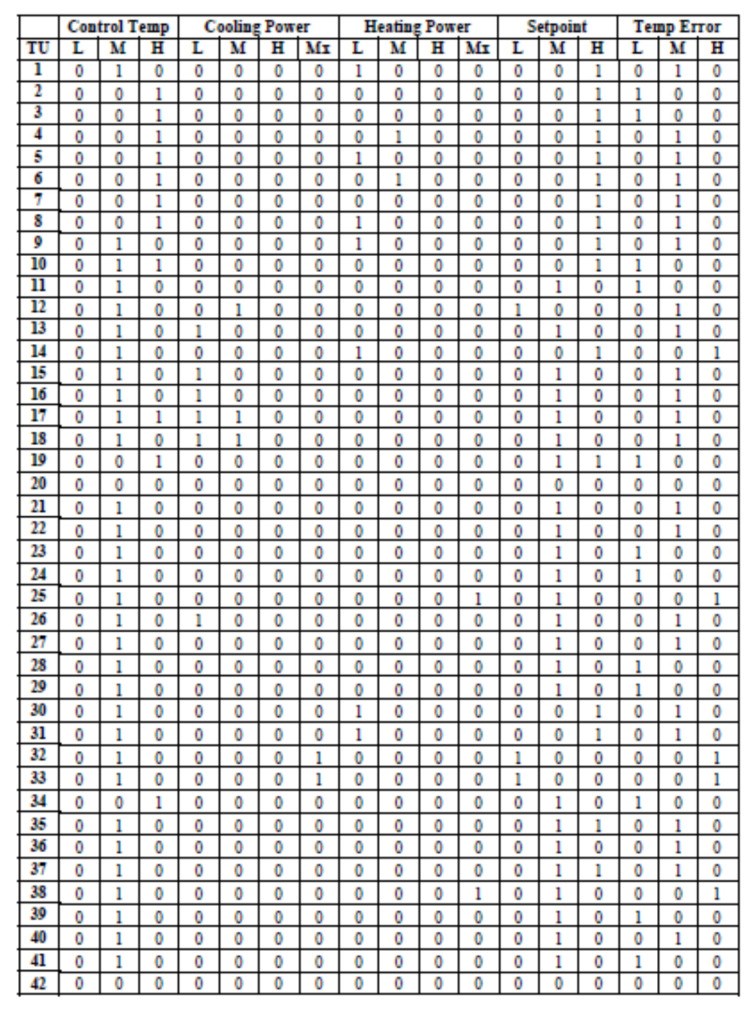
Incidence matrix for the Terminal Units (TUs) on a floor for one 15 min interval. A 1 in a cell indicates that the TU exhibits the behavior represented by the column during that interval.

Figure 5 shows the incidence matrix created for all of the TUs for a fifteen-minute time interval for a particular floor in the building. The rows indicate the TU numbers (there are 42 TUs in total for the given floor) and the columns indicate the different quantized states that can be exhibited by a TU. For example, Control temperature could be L-low, M-medium or H-high, Cooling Power consumed could be L-low, M-medium, H-high or Mx-maximum. Similarly, different states are shown for other variables: heating power, setpoint and temperature error. The incidence matrix cell contains a 1 if a particular TU exhibits the states (behavior) indicated by the column name or a 0 otherwise during the 15 min time interval. 

#### 3.3.2. Creation of Shared Face Matrices

**Figure 6:**
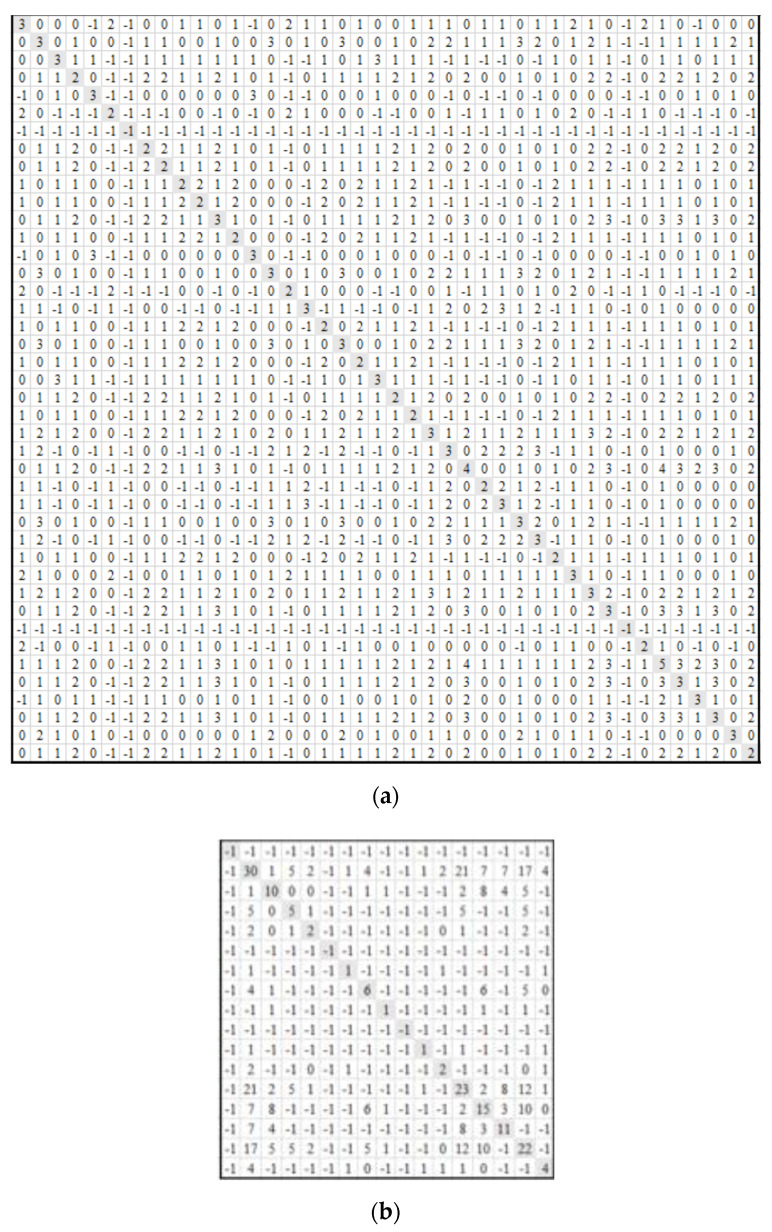
(**a**) TU shared face matrix (**b**) State shared face matrix. Each number in the matrix represents the dimension of the face (number of common vertices-1) that each TU or state shares with other TUs or states and 1 indicates no shared face, a face of 0 indicates a single vertex in common between pairs of simplices.

In general, if two TUs share q + 1 states, they are said to share a q-face or referred to as being q-near. The q-nearness of all pairs of TUs is examined by constructing a shared face matrix, which is a symmetrical matrix. Figure 6a represents the TU shared face matrix and the entries in the matrix indicate the common states between a pair of TUs, while the diagonal entries represent the dimensionalities of the TU simplices. Similarly, Figure 6b represents the state shared face matrix. These diagonal face matrices are created as per the matrix computations specified in Section 2.3 by left multiplication of the incidence matrix with its transpose and subtracting 1 from each entry to give the TU shared face matrix, and right multiplication of the incidence matrix with its transpose and subtracting 1 to give the state shared face matrix It can be further observed that the dimension of the TU simplicial complex is 5 i.e., a TU exhibits maximum 6 states, while the dimension of the state simplicial complex is 30 i.e., a state is exhibited by maximum 31 TUs. This dimensionality represents the maximum value of the diagonal elements. 

#### 3.3.3. Creation of q-Connectivity Lists and Q-Vectors

In addition to the direct connections between all pairs of TUs, there can be indirect connections too. These connections or q-connectivity (q-chains) exist at different dimensions. It is important not to confuse q-connectivity with q-nearness since a pair of simplices may be q-connected, but not q-near. On the other hand, q-nearness does imply q-connectivity. Q-Analysis helps reveal these q-connectivity lists. The q-connectivity lists enable examination of the global system structure by counting the number of equivalence classes at each q-level. This information about the number of equivalence classes or components at each q-level can be represented using structure vectors called Q-vectors.

**Figure 7:**
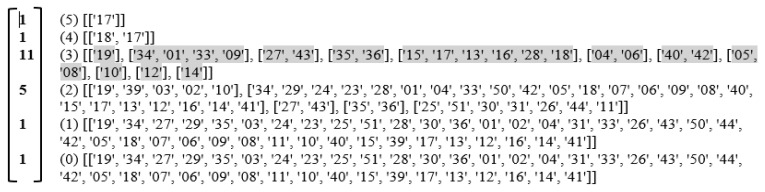
The q–connectivity lists and corresponding TU Q-vector. The left-hand column is the TU Q-vector i.e., number of components at each q-level, the number in the parentheses indicates the q-level, the square brackets indicate the connected components.

**Figure 8:**
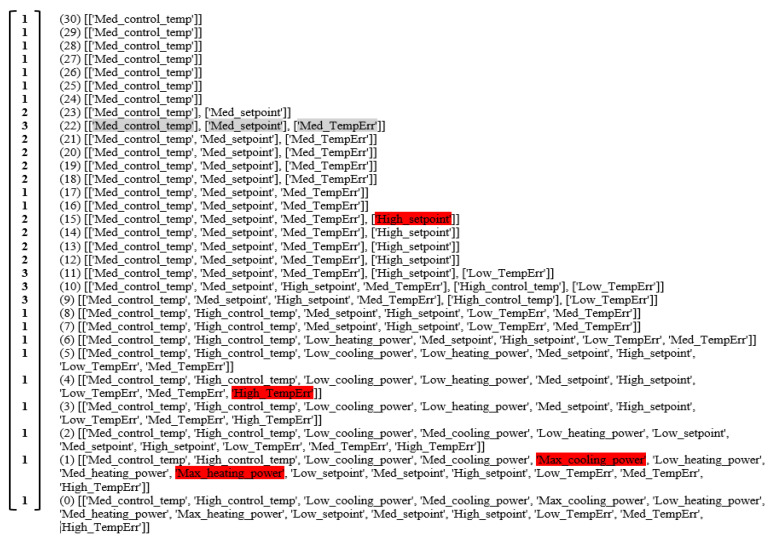
The q–connectivity list and corresponding state Q-vector. The left-hand column is the state Q-vector, the number in the parentheses indicates the q-level, the square brackets indicate the connected components. The highest q-value (30) shows for this time interval 31 TUs were in state medium control temperature, although only 24 TUs had medium set point (q = 23), while 16 TUs had a high set point (q = 15). A total of 12 TUs had low temperature error, while 5 TUs had high temperature error, 2 TUs were at maximum cooling power and maximum heating power. Using the incidence matrix as shown in Figure 5, it is possible to drill down to see which TUs are exhibiting anomalous behavior in an automated manner.

The q-connectivity lists for the various TUs and the corresponding TU Q-vector can be obtained following the Q-Analysis and are shown in Figure 7. Large entries in the Q-vector indicate that the structure is a fragmented or disconnected one. For example, at q-level = 3, there are 11 TU equivalence classes or components. Similarly, the q-connectivity lists for the various states and the corresponding state Q-vector can be obtained following the Q-Analysis and are shown in Figure 8. It is to be noted that brackets enclose each component and an examination of the structure within a component may be important and gives a perspective on local structure.

#### 3.3.4. Data Visualization

Once the TU and state Q-vectors have been computed using Q-Analysis, the Q-vector magnitudes can be visualized and compared against one another to give an insight into the TU behaviors on different floors. A higher TU Q-vector magnitude indicates a larger number of q-equivalence classes and hence more TUs engaging in an increased number of different states. Higher TU/state Q-vector magnitudes correspond to a higher degree of TU/state activity and hence more dynamic floor behavior, whereas for lower TU/state Q-vector magnitudes, the TUs/states engage in less activity and hence indicate a more ordered floor behavior.

**Figure 9:**
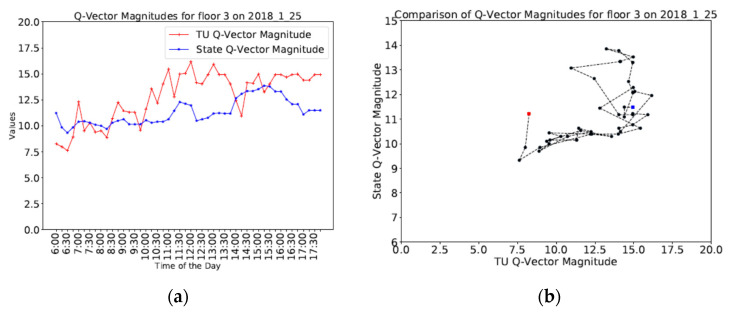
(**a**) Temporal variation of Q-vector magnitudes (**b**) Scatterplot of TU and state Q-vector magnitudes.

Different data visualizations are carried out for the TU and state Q-vector magnitudes for TUs belonging to a single building floor and are shown in Figure 9a,b. In Figure 9a, the TU and state Q-vector magnitudes are plotted to identify the temporal behavior during a single day, but the distinct phases are less clear, while in Figure 9b the scatterplot of the TU and state Q-vectors is shown. The comparison of many such plots show distinct phases of behavior, but the temporal aspects are lost. Nevertheless, some of the temporal aspects can still be captured by adding a line plot to the scatterplot showing the movement from one time instant to another and distinguishing the start and finish endpoints (red and blue squares respectively).

## 4. Results and Discussion

In this section, the results from the Q-Analysis using BEMS data are presented. Analysis was carried out across a number of days taken from different months in a year. However, for presentation, a series of days have been chosen from separate months of the year—25th July 2017, 25th October 2017, 25th January 2018 and 25th April 2018 across the 17 different building floors. In order to understand the seasonal variation of TU behaviors, data from a summer (25th July 2017) as well as a winter day (25th January 2018) has been examined in further detail and the key results and findings are presented in the following subsections.

### 4.1. Daily and Seasonal TU Behaviors

The three key findings that emerged from the analysis of different TU and state Q-vector magnitude plots generated across different days from different seasons from different floors are as follows:

1. There is no correlation of TU behavior between floors.
2. TU behavior is not dependent on the time of day or time of year.
3. The floors may exhibit distinct phases of behavior throughout the day at any time of the year.

**Figure 10:**
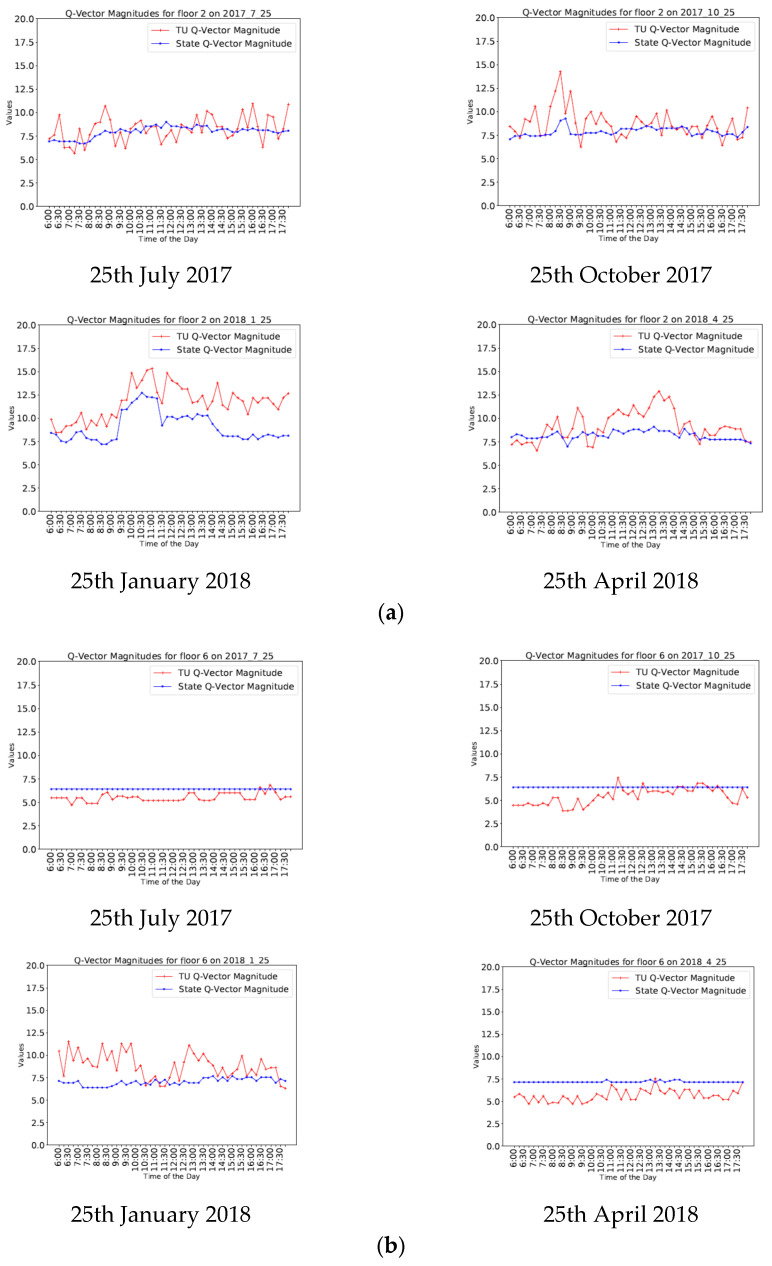

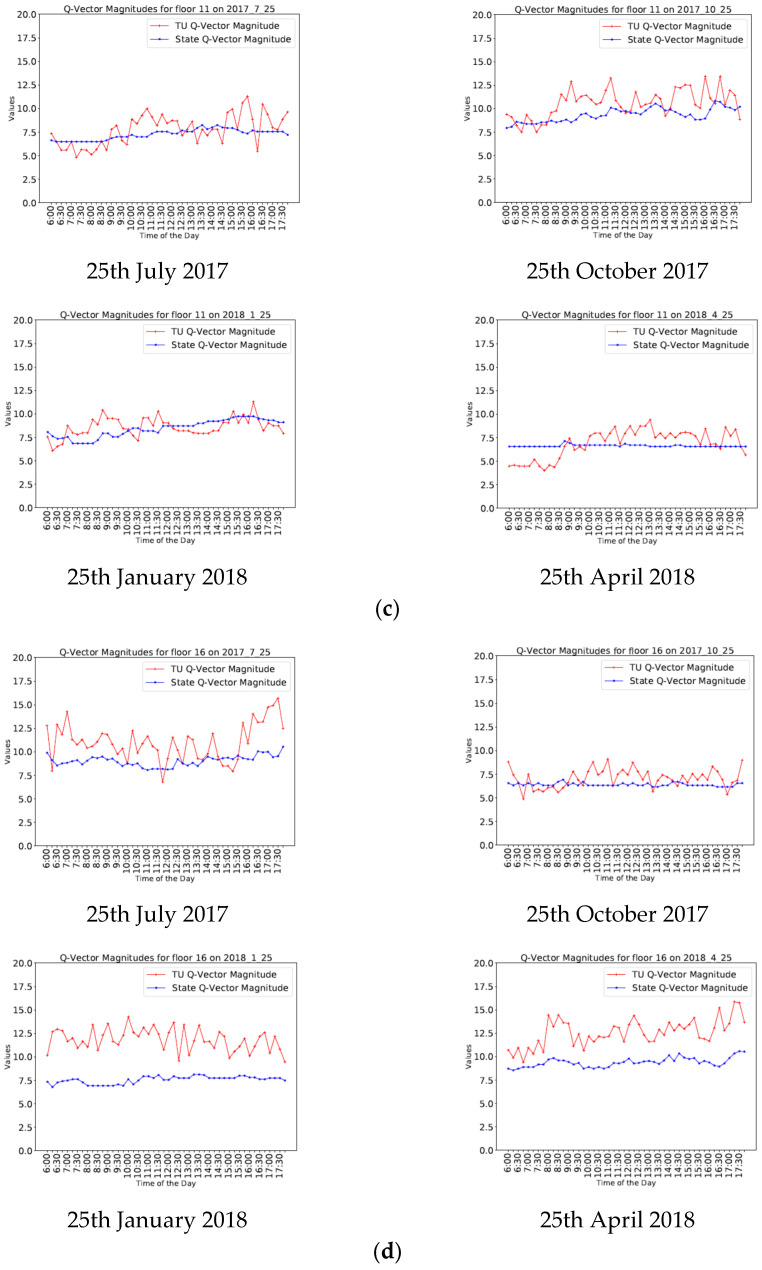
Graphs depicting the variation of TU Q-vector magnitudes and state Q-vector magnitudes on four different days across different seasons for (**a**) Floor 2 (**b**) Floor 6 (**c**) Floor 11 (**d**) Floor 16.

Figure 10 shows the graphs depicting the temporal variation of TU and state Q-vector magnitudes on different floors during four different days chosen from different times of the year as described above. These Q-vector magnitudes reveal very little hourly, daily or seasonal pattern information, but do make it evident that in general, there is far more variation in behavior between the floors. Individual floors can show very different behavior on different days, but these differences are not generally related to the time of year. This suggests that occupancy usage is a far more significant factor than quotidian variation.

### 4.2. Analysis of Q-Vector Behavior

As mentioned in the previous section, the temporal variation of Q-vector magnitudes depicted distinct behaviors across floors and hence was further examined. Following the analysis, it was found that some of the floors depict an ordered and stable behavior, whereas some of the floors showed a more dynamic Q-vector magnitude variation. It was also noticeable that initially mild behavior could become dynamic later during the day and vice versa. The variation is the same across both the TU and state Q-vector magnitudes. As mentioned earlier in Section 3.3.4, a high value of TU Q-vector magnitude indicates that multiple TU q-equivalence classes are in different states throughout the day, whereas a high value of the state Q-vector magnitude is a result of multiple state q-equivalence classes.

**Figure 11:**
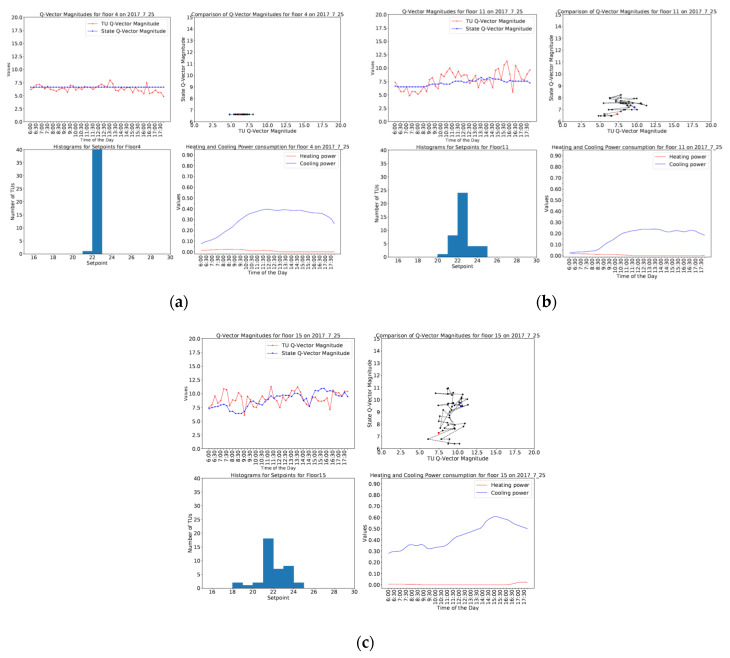
Graphs depicting the variation of TU Q-vector magnitudes and state Q-vector magnitudes, Scatterplot of TU and state Q-vector magnitudes, Set point histogram, Heating and cooling power consumption for different behaviors on (**a**) Floor 4 (**b**) Floor 11 (**c**) Floor 15 on 2017.7.25 (summer day).

**Figure 12:**
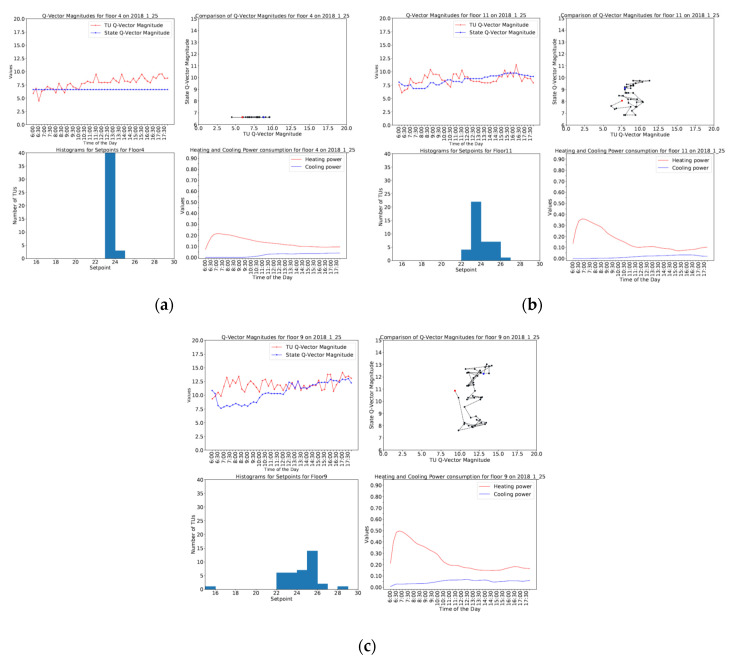
Graphs depicting the variation of TU Q-vector magnitudes and state Q-vector magnitudes, Scatterplot of TU and state Q-vector magnitudes, Set point histogram, Heating and cooling power consumption for different behaviors on (**a**) Floor 4 (**b**) Floor 11 (**c**) Floor 9 on 2018.1.25 (winter day).

Figure 11 shows the three different behaviors exhibited by different floors on a summer day. It is seen in Figure 11a that floor 4 has a stable behavior with almost no variation in state Q-vector magnitude and very little variation in TU Q-vector magnitude; while in Figure 11b, floor 11 shows mild dynamic behavior with little variation in state Q-vector magnitude in addition to slightly more variation in TU Q-vector magnitude. Figure 11c shows dynamic behavior displayed by floor 15 with higher variation in both TU and state Q-vector magnitude. Similarly, Figure 12 shows stable behavior for floor 4, mild dynamic behavior for floor 11 and dynamic behavior for floor 9 on a winter day.

Further, the scatterplots of the TU and state Q-vector magnitudes show that different floor behaviors are quite distinct. Different floor behaviors were observed that could be categorized as follows with respect to either the TU or state Q-vector magnitudes.

1. Ordered floor behavior is minimal variation in state Q-vector magnitude with both low average TU and state Q-vector magnitudes.
2. Mild dynamic floor behavior is greater variation in either the TU or state Q-vector magnitudes or both.
3. Dynamic floor behavior is the greatest variation in either the TU or state Q-vector magnitudes or both.

It is also noted that these scatterplots occupy different positions in the phase space. The different behaviors mentioned above can be visualized in Figure 11 and Figure 12. During the day, a floor could transition from one behavior to another. Figure 11 shows the floor behaviors on a summer day with a straight-line scatterplot for floor 4, mild dynamic behavior for floor 11 with a scatterplot in the bottom-left quadrant of the phase space, dynamic behavior for floor 15 with a scatterplot extending to the top-right quadrant of the phase space. Similarly, Figure 12 shows the floor behaviors on a winter day with stable behavior for floor 4, mild dynamic behavior for floor 11, dynamic behavior for floor 9 in terms of scatterplots between the TU and state Q-vector magnitudes.

### 4.3. Correlation with Set Point and Power Behaviors

It was concluded with building service experts that the set point and heating/cooling power are the two key factors affecting the TU behaviors. Hence, the floor behaviors were correlated with the set point and power consumption behaviors on each of the floors. Histograms were generated for all the TU set points on a day and similarly, total heating and cooling power consumed by all the floor TUs was computed and plotted.

It was observed that the floors showing ordered behavior had tightly bound set points, whereas the floors with dynamic behaviors had greater set point variation and relatively higher power consumption typically with a mixture of both heating and cooling. This is evident from the set point distributions and total heating or cooling power consumption shown for different floors on both summer and winter days in Figure 11 and Figure 12. In case of ordered floor behavior, it is observed that most of the TUs have the same set point, whereas in the case of mild floor behavior, there is slightly more variation in set points with a maximum set point variation shown by floors with dynamic behavior. This leads to the conclusion that the distribution of set points is a significant factor in TU behavior and power consumption, leading to different floor behaviors.

## 5. Conclusions and Future Directions

This paper set out to apply Q-Analysis as a data summarization technique for large IoT datasets to reveal intrinsic shape-based data characteristics. The insight on individual floor behaviors was not evident in previous work using the unsupervised learning methods. Q-Analysis provided a means for summarizing global behavior—extracting data from a set of sensors and describing the data in a simpler manner. Using Q-Analysis on BEMS data has revealed a number of useful system characteristics. The novel extracted feature of TU and state Q-vector and their corresponding visualization, namely the TU and stated Q-vector magnitude scatterplot, provides a valuable snapshot for depicting the relationships between two datasets. This can further be applicable to any domain, where measures can be suitably discretized. Based on the Q-Analysis of the BEMS data, it was possible to identify distinct floor behaviors easily in a visual manner. TU and state Q-vector magnitude scatterplots across different days and seasons can provide a single snapshot to get an insight into the floor behaviors. Furthermore, it has been shown that the floor behaviors are not dependent on time and season of the year and in fact a relationship between set point distribution and floor behavior has been discovered. Also, the q-connectivity lists can provide a mechanism to drill down to pinpoint the TUs that are responsible for anomalous behaviors.

The computational overhead of Q-Analysis is light. The incidence matrix calculation incurs greater computational load, which typically has a few hundred columns and rows and are relatively sparse, for which efficient algorithms are well established, making this technique ideal for a first cut analysis of a wide variety of large IoT sensor data. Nevertheless, there are some limitations to the Q-Analysis technique. The choices at the data preprocessing stage, namely the variable selection and their appropriate discretization ranges and temporal intervals, are crucial. Lack of standards in building energy systems exacerbated issues and the need for domain experts is essential in this regard. For instance, in the current study the set point settings were assumed to be between 21 °C and 24 °C based on advice from the building engineers, as opposed to experimentation based on empirical averages.

There are number of further aspects of Q-Analysis that can be exploited as a part of future work. A key area of Q-Analysis that has not been examined in this paper relates to eccentricity, which would further help to identify the local TU structure and could be particularly helpful in faulty TU identification. The work has been limited to the application at hand i.e., BEMS data analysis, though the Q-Analysis can prove to be very useful in other application domains, particularly where flow data is available. For instance, in this work, it would have been really useful to have more information on floor usage and occupancy data, say for example, data from the elevators or mobile phone presence could be really useful. Thus, this work will be very impactful for future smart building applications and with a suitable software tool support for each stage of the methodology, this technique could be applied to a wide range of IoT scenarios as a first cut knowledge discovery technique to identify key systems variables and their relationships.
